# Long Non-Coding NONRATG001910.2 Promotes the Proliferation of Rat Mesangial Cell Line HBZY-1 Through the miR-339-3p/CTNNB1 Axis

**DOI:** 10.3389/fgene.2022.834144

**Published:** 2022-04-28

**Authors:** Jiarong Gao, Xiaoli Zhu, Hao Chen, Hui Jiang, Miaomiao Shi, Liangbing Wei, Xiujuan Qin

**Affiliations:** ^1^ Department of Pharmacy, The First Affiliated Hospital of Anhui University of Chinese Medicine, Hefei, China; ^2^ Anhui Province Key Laboratory of Chinese Medicinal Formula, Hefei, China; ^3^ College of Pharmacy, Anhui University of Chinese Medicine, Hefei, China

**Keywords:** chronic glomerulonephritis, long non-coding RNA, NONRATG001910.2, miR-339-3p, CTNNB1

## Abstract

Chronic glomerulonephritis (CGN) is one of the leading causes of end-stage renal disease (ESRD). A growing body of literature emphasizes the important role of long non-coding RNAs (lncRNAs) in the development and progression of the disease. However, the function of NONRATG001910.2 in the development of CGN was not well understood. This research aimed to investigate the effect of NONRATG001910.2 on CGN and revealed its potential molecular mechanisms. In this work, the expression of NONRATG001910.2 was detected by quantitative real-time polymerase chain reaction (qRT-RCR) in cell lines. We found that NONRATG001910.2 was significantly up-regulated in lipopolysaccharide (LPS) induced cells. High NONRATG001910.2 levels were associated with the development of CGN. In addition, NONRATG001910.2 knockdown inhibited cell proliferation and cell cycle. At the same time, we found that up-regulation of microRNA-339-3p (miR-339-3p) abrogated the biological roles of NONRATG001910.2 up-regulation. Moreover, the knockdown of CTNNB1 can upregulate miR-339-3p expression, thereby inhibiting cell proliferation. In conclusion, these results demonstrated that NONRATG001910.2 in LPS-stimulated rat mesangial cell line HBZY-1 (HBZY-1) by targeting miR-339-3p, which subsequently promotes the expression of CTNNB1, and suggested that NONRATG001910.2 may be a potential biomarker.

## Introduction

Kidney disease represents one of the most important non-communicable diseases worldwide. Chronic kidney disease (CKD) has become a major global health challenge for its growing prevalence and disease burden over the decades and the number of CKD cases has been estimated to be over 697 million worldwide in 2019 (Liang et al., 2022). Chronic glomerulonephritis (CGN) is the most common form of primary glomerular disease and the cause of end-stage renal disease in patients with chronic kidney disease (Murkamilov et al., 2018; Hu et al., 2020; Jia et al., 2021). The main clinical features are proteinuria, hypertension, oedema and haematuria (Li et al., 2018). The research group started from miRNA to study the molecular mechanism of CGN in the early stage (Wang et al., 2020), and this study is based on the previous research, looking for the upstream genes of miRNA, and further study the molecular mechanism of CGN. Therefore, it is important to study the molecular characteristics of CGN in depth to help understand the pathogenesis of CGN and develop new diagnostic markers.

Approximately 2% of the human genome encodes proteins, while the majority of the genome is transcribed into non-coding RNA (ncRNA) (Cheng et al., 2019; Wang et al., 2019). Depending on the length of the transcript, ncRNA can be classified as small ncRNA (≤ 200 nt) or long ncRNA (> 200 nt) (Li et al., 2019; Liu et al., 2020). LncRNA lacks a complete open reading frame and does not have a protein-coding function. An increasing number of studies have shown that lncRNAs play important roles in a variety of biological processes through different mechanisms, including epigenetic modification, chromatin remodeling, and post-transcriptional or transcriptional (Ponting et al., 2009; Tsai et al., 2010; Nagano and Fraser, 2011). Recently, with the advancement of technology, more and more aberrantly expressed lncRNAs have been identified in kidney diseases (Ignarski et al., 2019). For example, increased expression of LncRNA CDKN2B-AS1 in diabetic nephropathy (DN) may regulate thylakoid cell proliferation and extracellular matrix accumulation and influence disease progression (Li Y. et al., 2020). NONRATG001910.2 is a lncRNA that is derived from chromosome 1:78843077-78844692. Our team previously studied the differential expression of LncRNAs in the renal cortex of CGN rats (Qin et al., 2019). We found that NONRATG001910.2 (Rat) and NONHSAG026091.2 (human) length coverage = 52%, *e*-value = 3.00E-135, bit score = 819, indicating NONRATG001910.2 (rat) and NONHSAG026091.2 (human) are conservative. The fold change of NONRATG001910.2 between the model group and the normal group is greater than 5, *p* = 0.0024, and the FPKM value can be up to 64. NONRATG001910.2 transcript length is 1,615 bp, which is less than 2000 bp. Sequence refinement of the NONRATG001910.2 transcript in the noncode database, and we choose NONRATG001910.2 for follow-up research. The ability of lncRNAs to influence cellular activity by targeting miRNAs or regulating signaling pathways during disease progression has led to the choice of starting with lncRNAs to investigate the mechanisms involved in CGN.

MIRNA is a small ncRNA molecule composed of about 22 nucleotides. miRNA inhibits the translation of messenger RNA (mRNA) by combining its seed sequence with the 3 ‘untranslated region (UTR) of mRNA, which leads to the degradation of mRNA. LncRNAs can act as post-transcriptional regulators, competitively inhibiting the function of miRNA and regulating gene expression, thus contributing to the development of disease, a mechanism known as the “competitive endogenous RNA (ceRNA)” hypothesis (Salmena et al., 2011). For example, SNHG16 upregulated CCND1 expression in diabetic nephropathy by competitively binding miR-141-3p, inducing proliferation and fibrosis (Jiang et al., 2020). LINC00467 knockdown inhibits cell proliferation but stimulates apoptosis via the miR-339-3p/IP6K2 axis in glioblastoma (Liang and Tang, 2020). Wnt/β-catenin signaling regulates cellular processes by regulating the expression of β-catenin protein, an important growth stimulator in the Wnt/β-catenin pathway, leading to cell proliferation (Jiang et al., 2021).

In the present study, we found that NONRATG001910.2 in a gain-of-function or loss-of-function state affected LPS-induced HBZY-1 proliferation and cell cycle progression. Furthermore, miR-339-3p is a target of NONRATG001910.2 and CTNNB1 is a downstream target of miR-339-3p. The NONRATG001910.2/miR-339-3p/CTNNB1 axis may be a novel finding in chronic glomerulonephritis.

## Materials and Methods

### Cell Culture and Transfection

Rat mesangial cell line (HBZY-1) was purchased from iCell Bioscience Inc (Shanghai, China). The cultured cell was maintained routinely in DMEM media (Hyclone, United States) supplemented with 10% fetal bovine serum (Gibco, AU) and incubated at 37°C in ahumidified atmosphere containing 5% CO2. Specific siRNA sequence with a certain interference effect was purchased from GenePharma (Shanghai, China). NONRATG001910.2 overexpression plasmid was obtained from GenePharma (Shanghai, China), where an empty vector (pcDNA3.1) was used as a negative control. Transfection was performed using Lipofectamine 2000 (Invitrogen, United States).

### qRT-PCR

Based on the manufacturer’s instructions, total RNA from HBZY-1 cells was drawn out with TRIzol reagent (Invitrogen, United States). Reverse transcription of lncRNA, miRNA and mRNA were performed using Prime Script RT Reagent Kit (Takara, JAP). After this, we performed real-time quantitative fluorescence PCR (qRT-PCR) using SYBR Green Master Mix (Novoprotein, Shanghai, China). The thermocycling conditions were as follows: Pre-denaturation at 95°C for 1 min, 40 cycles at 95°C for 20 s and 60°C for 1 min. The relative expression levels of lncRNA, miRNA and mRNA were separately calculated with the 2−(^ΔΔ^Ct) method (Livak and Schmittgen, 2001). The U6 was used as the internal reference of miR-339-3p, while β-actin was the internal reference of lncRNA and mRNA. The 2-(^ΔΔ^Ct) method was used for relative quantification as follows: ^Δ^Ct _test = Ct (target, test)—Ct (reference, test), ^Δ^Ct _control = Ct (target, control)—Ct (reference, control), 2-(^ΔΔ^Ct) = 2-(^Δ^Ct _test—^Δ^Ct _control). We did 9 repetitions of this experiment. The primers for qRT-PCR were depicted in Supplementary Table S1.

### Fluorescence *in Situ* Hybridization

Fluorescence *in situ* hybridization (FISH) was performed on HBZY-1 cells to determine the subcellular location of NONRATG001910.2. The cell specimens were placed in 4% paraformaldehyde for 20 min, and then incubated overnight with lncRNA FISH probe diluted in hybridization solution at 37°C. Next, the hybridization solution was washed off. The cell specimens were stained with DAPI for 10 min at room temperature, and finally observed with an inverted fluorescent microscope (Nikon, Japan).

### Dual Luciferase Reporter Assay

Mutant luciferase reporter vectors NONRATG001910.2 MUT and CTNNB1 MUT were generated by mutating the predicted seed site to nullify miR-339-3p binding. The constructed luciferase reporter vector and miR-339-3p mimics or microRNA-negative control (miR-NC) were transfected into HBZY-1 cells for 48 h and luciferase activity was assayed using a dual luciferase reporter assay system (Promega, United States). The miR-NC is a generic sequence with no definitive species information.

### Cell Proliferation Assay

Cell proliferation was assessed according to instructions provided with a Cell Counting Kit-8 (Bioss, Beijing, China). Briefly, cells were collected and transferred into 96-well flat-bottomed microplates. Next, the cells were incubated with 100 μl of CCK8 solution for 4 h, after which, the absorbance at 450 nm was recorded. We did three replications of the CCK8 experiment. The cell viability was calculated as follows: Cell viability = [(OD value of treatment group—OD value of blank group)/(OD value of control group—OD value of blank group)] × 100%.

### Nucleocytoplasmic Fractionation

Cytoplasmic and nuclear RNA were separated and purified utilising the Cytoplasmic and Nuclear RNA Purification Kit (Norgen biotek, CAN) in accordance with the manufacturer’s instructions. Then, qRT-PCR was conducted to detect the level of NONRATG001910.2 extracted from each of the fractions. GAPDH and U6 were, respectively, used as cytoplasmic and nuclear control. The experiment was repeated nine times.

### Western Blot Analysis

Each group of cells was collected and lysed in RIPA buffer (Beyotime, Shanghai, China) containing protease inhibitors. The protein concentration was measured using the bicinchoninic acid (BCA) protein assay Kit (Beyotime, Shanghai, China). Equal amounts of extracts were added for sodium dodecyl sulphate-polyacrylamide gel (SDS-PAGE) to separate proteins (Beyotime, Shanghai, China). Then, gels were transferred to a polyvinylidene difluoride (PVDF) membranes (Millipore, United States). The membranes were blocked using 5% skimmed milk powder. Then, using the following primary antibodies at 4°C overnight: anti-β-catenin (Cell Signaling, United States, 1:1000), anti-c-Myc (Abcam, United Kingdom, 1:1000), anti-CyclinD1 (Bioss, Beijing, China, 1:500). After incubation with secondary HRP antibody (Zsbio, Beijing, China) for 2 h at room temperature, the signal on the membrane was observed using an enhanced chemiluminescence kit (Thermo, United States). This experiment was repeated three times. Results were analysed using ImageJ software. With β-actin as internal reference, the relative expression of proteins was detected and expressed as the ratio of the gray values between the target band and internal reference band.

### Flow Cytometry Assay

Apoptosis rates and cell cycle distribution were measured by flow cytometry for each group of cells. As for cell cycle analysis, it was performed using a Cell Cycle and Apoptosis Analysis Kit (Biosharp, Shanghai, China), according to the manufacturer’s protocols. Kaluza was used to analyse cell cycle distribution. For apoptosis assays, the Annexin V-FITC/PI Apoptosis Kit (MultiSciences, Shanghai, China) was used according to the manufacturer’s protocol. Cells were collected and washed twice with a pre-cooled PBS buffer. Five hundred microliters of 1xBinding Buffer, 5 µl of Annexin V-FITC and 10 µl of PI were added to each sample for 5 min at room temperature in the dark. All flow cytometry experiments were repeated three times.

### Immunofluorescence Assay

Cells grown on coverslips were fixed in 4% paraformaldehyde for 20 min and then permeabilized by incubation in 0.3% Triton X-100 for 20 min at room temperature. After blocking, cells were immunostained and incubated for 1 h with a primary antibody. The antibodies were diluted as follows: β-catenin, 1:300; c-Myc, 1:200 and CyclinD1, 1:200. After subsequent washes, the sections were incubated with the appropriate secondary antibody for 30 min at room temperature, and the nuclei were stained with DAPI solution 5 min at room temperature. Immunofluorescence experiments were repeated three times. Fluorescence images were analyzed by JD801 for average optical density.

### Cell Ultrastructure

The cells of each group were collected, fixed with 2.5% glutaraldehyde and 1% ozone acid, dehydrated with acetone and injected with epoxy resin embedding agent. Finally, the samples were sliced and stained, and the ultrastructure of the cells was observed through a transmission electron microscope.

### Statistical Analysis

Statistical analysis was performed using SPSS 25.0 statistical software package. All data is expressed as mean ± standard deviation (SD). In order to compare the differences between two or more groups, *t*-test or one-way analysis of variance (ANOVA) was used to analyze the differences. All statistical tests were 2-sided, where a value of *p* < 0.05 was considered statistically significant.

## Result

### NONRATG001910.2 Promotes the Progression of Chronic Glomerulonephritis

**FIGURE 1:**
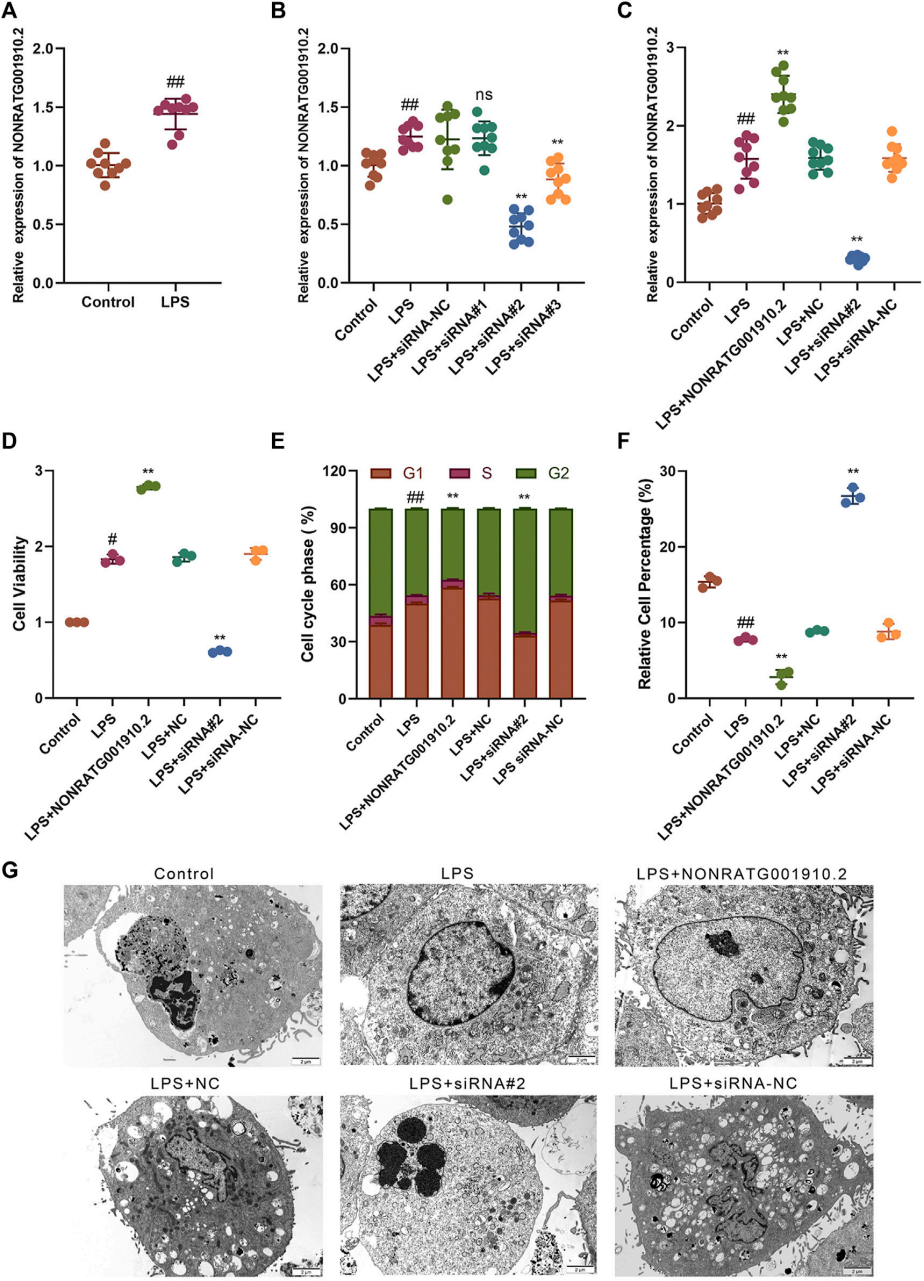
NONRATG001910.2 promotes HBZY-1 cell growth and cell cycle progression and inhibits cell apoptosis. **(A)** Relative expression level of NONRATG001910.2 in control group and LPS stimulated group. **(B)** qRT-PCR detection of relative expression level of NONRATG001910.2 in siRNA interference vector. **(C)** Expression of NONRATG001910.2 in overexpressed and knockdown states by qRT-PCR. **(D)** CCK-8 assay was used to detect changes in the proliferation of HBZY-1 cells in the gain-of-function or loss-of-function state of NONRATG001910.2. **(E,F)** Flow cytometric detection of cell cycle distribution and apoptosis in HBZY-1 cells in the gain-of-function or loss-of-function state of NONRATG001910.2. **(G)** Transmission electron microscope observation of cell ultrastructure. ^#^ *p* < 0.05 and ^##^ *p* < 0.01, compared with the control group. ^**^ *p* < 0.01, compared with the LPS stimulated group.

To investigate the effect of NONRATG001910.2 on the progression of chronic glomerulonephritis, the qRT-PCR assay was performed to detect the expression of NONRATG001910.2. The expression of NONRATG001910.2 in LPS-induced HBZY-1 cells was significantly higher than that in the control group (Figure 1A). Three small interfering RNAs directed against NONRATG001910.2 were transfected into LPS-induced cells. It was found that NONRATG001910.2 was significantly reduced after transfection with siRNA^#^2, so siRNA^#^2 was selected for the subsequent experiments (Figure 1B). The expression of NONRATG001910.2 in the cells was detected after transfection of siRNA^#^2 as well as the overexpression plasmid, respectively, and it was found that the expression of NONRATG001910.2 was significantly affected (Figure 1C). CCK8 assay was performed to detect cell proliferation. We transferred siRNA^#^2 and over-expression plasmid into LPS-stimulated HBZY-1 cells separately to detect cell proliferation and found that the NONRATG001910.2 plasmid-transfected group showed significant proliferation compared to the LPS-stimulated HBZY-1 cells (Figure 1D). Flow cytometry of the LPS-stimulated HBZY-1 cell cycle revealed that knockdown of NONRATG001910.2 blocked the cell cycle in the G2 phase (Figure 1E, Supplementary Figure S1A). Meanwhile, we detected apoptosis by flow cytometry using the FITC/PI double-staining method (Figure 1F, Supplementary Figure S1B). The results showed that the rate of apoptosis was increased after the knockdown of NONRATG001910.2 compared to LPS-stimulated cells. In contrast, when the NONRATG001910.2 plasmid was transfected in LPS-stimulated cells, over-expressing NONRATG001910.2, the rate of apoptosis was significantly reduced. Ultrastructural observation of the cells by transmission electron microscopy revealed after overexpression of NONRATG001910.2 in LPS-stimulated cells, compared with the LPS-stimulated group, the overall cell morphology and structure were more normal, the nucleus was oval, the nuclear membrane was slightly tortuous, the nuclear chromatin was uniform, the nuclear membrane space was normal, and there was no apoptosis phenomenon. After knockdown of NONRATG001910.2 in LPS-stimulated cells, multiple apoptotic bodies have been generated compared to the LPS-stimulated group (Figure 1G).

### NONRATG001910.2 is the Sponge For miR-339-3p

**FIGURE 2:**
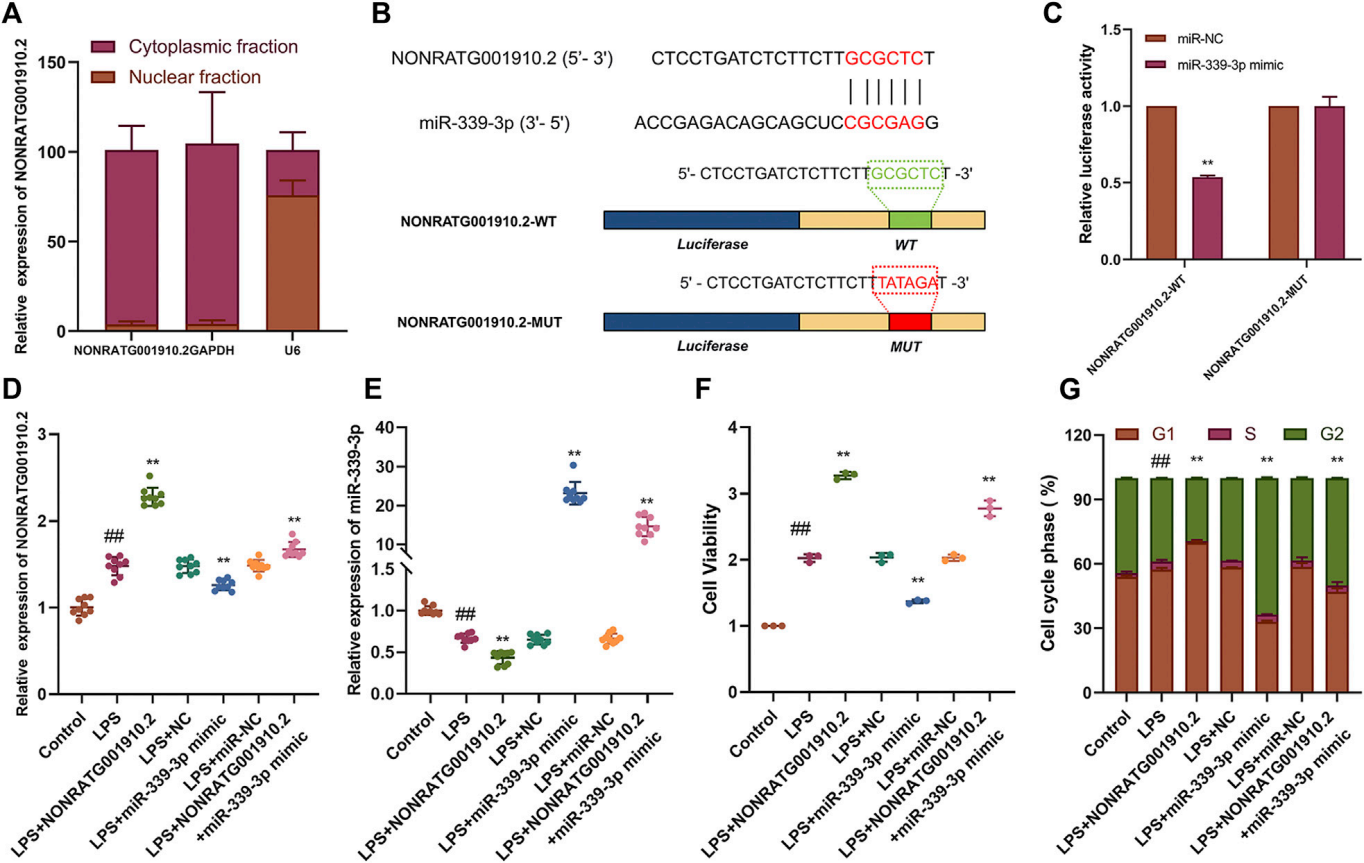
NONRATG001910.2 functions as molecular sponge for miR-339-3p. **(A)** NONRATG001910.2 abundant in the cytoplasm of HBZY-1 cells. GAPDH and U6 were used as positive controls in the cytoplasm and nucleus, respectively. **(B)** Schematic graph illustrating the mutation of potential binding sites between NONRATG001910.2 and miR-339-3p. **(C)** 293T cells that were co-transfected with miR-339-3p mimics or miR-NC and WT or MUT NONRATG001910.2 vector were measured for luciferase activity. HBZY-1 cells after LPS induction with miR-339-3p mimic or miR-NC transfected with NONRATG001910.2 gene overexpression were subjected to **(D,E)** qRT-PCR, **(F)** CCK-8, **(G)** cell cycle to determine NONRATG001910.2, miR-339-3p, cell proliferation and cell cycle progression. ^##^ *p* < 0.01, compared with the control group. ^**^ *p* < 0.01, compared with the LPS stimulated group.

LncRNAs can act as a “sponge” for different miRNAs in their various biological functions. NONRATG001910.2 was confirmed to be predominantly located in the cytoplasm (Figure 2A, Supplementary Figure S2A). Our team used high-throughput transcriptome sequencing technology to screen 40 differential miRNAs in the pathogenesis of CGN in the early stage. Through bioinformatics prediction and analysis, it was found that NONRATG001910.2 has the highest binding degree to miR-339. However, through preliminary experiments, it was found that miR-339-5p has no binding site with NONRATG001910.2. We proved that NONRATG001910.2 binds to miR-339-3p by predicting the binding sites of the two and by experimental verification. To explore the binding ability of NONRATG001910.2 to miR-339-3p, luciferase reporter vectors NONRATG001910.2-WT and NONRATG001910.2-MUT were constructed and transfected into LPS-stimulated HBZY-1 cells (Figure 2B). Dual-luciferase reporter analysis showed that overexpression of miR-339-3p resulted in reduced luciferase activity in the NONRATG001910.2-WT group, while the NONRATG001910.2-MUT group had little effect on luciferase activity (Figure 2C). When NONRATG001910.2 overexpression vector was transfected into LPS-induced HBZY-1 cells, the RNA level of NONRATG001910.2 increased and the level of miR-339-3p RNA decreased, promoting the proliferation of HBZY-1 cells and decreasing in cells in the G2 phase of the cell cycle (Figures 2D–G, Supplementary Figure S2B). In addition, co-transfection of NONRATG001910.2 and miR-339-3p into cells partially eliminated the effect of NONRATG001910.2 on promoting cell proliferation, resulting in a significant increase in cells entering the G2 phase.

### CTNNB1 is A Novel Target of miR-339-3p

**FIGURE 3:**
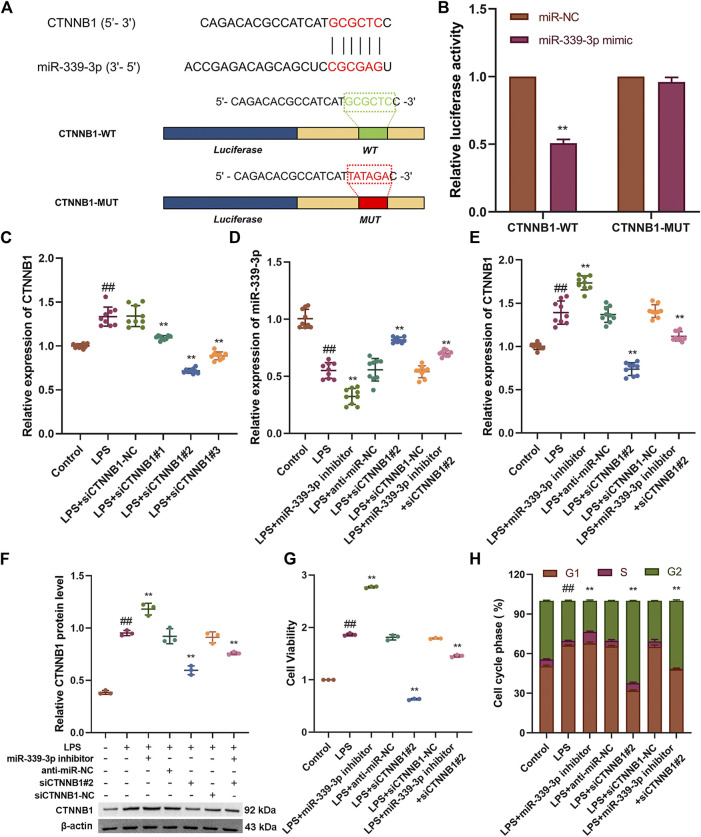
CTNNB1 knockdown attenuates the role of miR-339-3p gene downregulation in LPS-stimulated cells. **(A)** Schematic representation of the site-specific mutation of CTNNB1 binding to miR-339-3p. **(B)** Luciferase reporter analysis shows that luciferase activity of CTNNB1 3′UTR is significantly inhibited by miR-339-3p mimics. **(C)** Screening of CTNNB1 small interference sequence by qRT-PCR. **(D–F)** qRT-PCR and WB assays were performed to detect the expression levels of miR-339-3p as well as CTNNB1. **(G)** CCK-8 and **(H)** cell cycle assays were performed to determine cell proliferation and cell cycle progression. ^##^ *p* < 0.01, compared with the control group. ^**^ *p* < 0.01, compared with the LPS stimulated group.

To verify the target association between miR-339-3p and CTNNB1, the luciferase reporter vectors CTNNB1-WT and CTNNB1-MUT were co-transfected with miR-339-3p mimic or miR-NC in HBZY-1 cells (Figure 3A). The results showed that in the CTNNB1-WT group, the luciferase activity was significantly lower in the miR-339-3p mimic group compared to the miR-NC group, while in cells transfected with CTNNB1-MUT, the luciferase activity in the miR-339-3p mimic group was not significantly changed compared to the miR-NC group (Figure 3B). After transferring three small interfering RNAs into the cells separately, it was found that the expression of CTNNB1 was significantly reduced in LPS-induced cell lines transfected with siCTNNB1^#^2, so siCTNNB1^#^2 was selected for the subsequent experiments (Figure 3C). In addition, miR-339-3p downregulation significantly increased the expression of CTNNB1 mRNA and protein levels in HBZY-1 cells, while CTNNB1 downregulation also affected the RNA levels of miR-339-3p (Figures 3D–F). Transfection of miR-339-3p inhibitor into LPS-induced HBZY-1 cells promoted cell proliferation, allowing fewer cells to remain in the G2 phase. When miR-339-3p inhibitor and siCTNNB1^#^2 were co-transfected into LPS-induced cells, siCTNNB1^#^2 reduced the effects of miR-339-3p inhibitor on the level of CTNNB1 and promoted cell proliferation, allowing more cells to enter the G phase (Figures 3G,H, Supplementary Figure S3).

### NONRATG001910.2 Acts Through the ceRNA Mechanism

**FIGURE 4:**
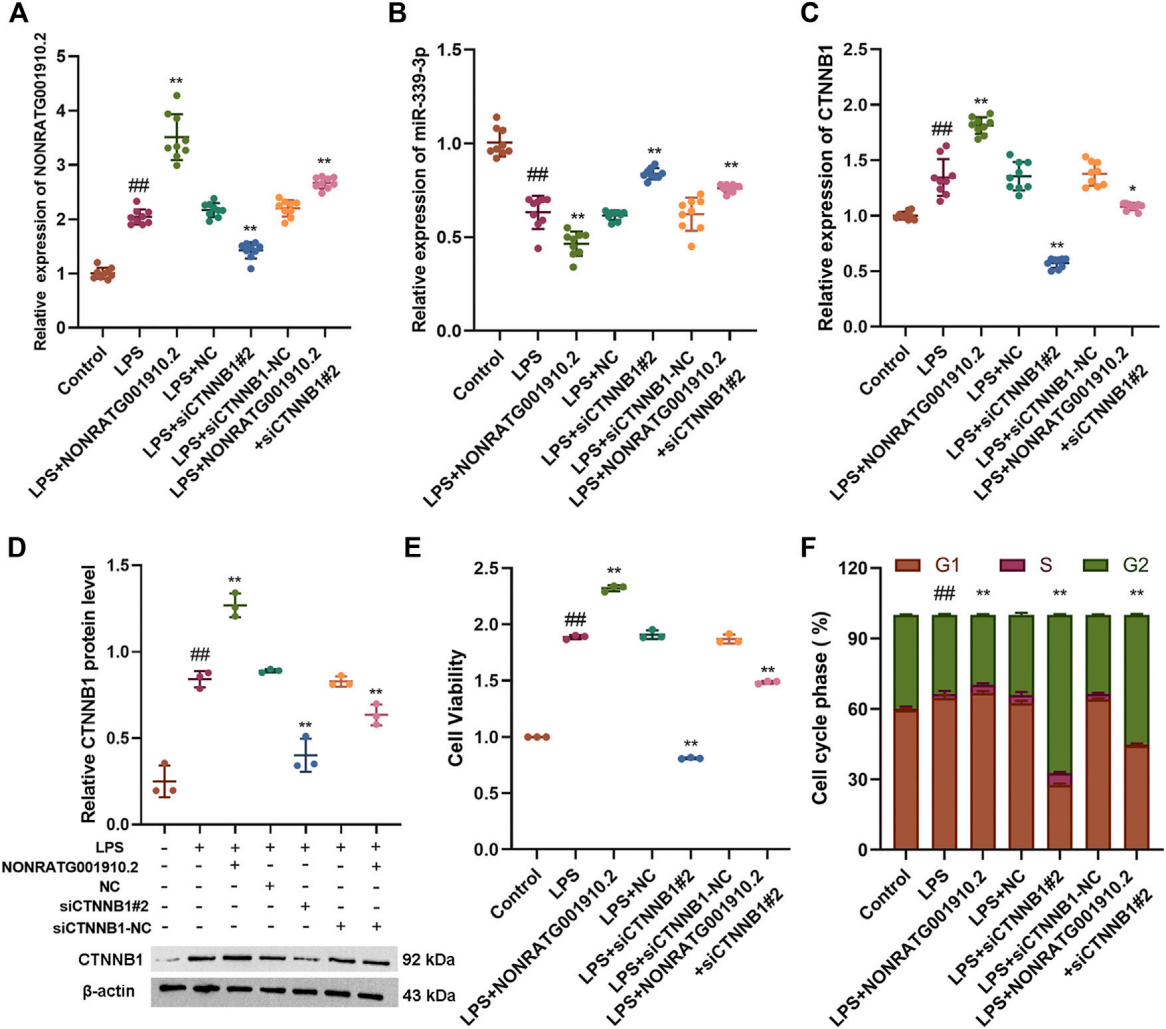
NONRATG001910.2 acts through the ceRNA mechanism. **(A,B)** qRT-PCR to detect the mRNA expression levels of NONRATG001910.2 and miR-339-3p **(C,D)** qRT-PCR and western blot were used to examine CTNNB1 expression at mRNA and protein levels. **(E)** CCK-8 and **(F)** cell cycle assays were performed to determine cell proliferation and cell cycle progression. ^##^ *p* < 0.01, compared with the control group. ^*^ *p* < 0.05 and ^**^ *p* < 0.01, compared with the LPS stimulated group.

To further explore whether NONRATG001910.2 could also function as a ceRNA mechanism. Based on the theory of ceRNA, it was found that overexpression of NONRATG001910.2 caused an increase in the mRNA and protein level expression of CTNNB1, a decrease in the RNA expression level of miR-339-3p, promoted cell proliferation and a decrease in the number of cells entering the G2 phase. When NONRATG001910.2 was co-transfected with siCTNNB1 into LPS-stimulated cells, the mRNA and protein levels of CTNNB1 expression were found to decrease compared to the LPS group, which eliminated the cell proliferation caused by NONRATG001910.2 over-expression and increased the number of cells lingering in the G2 phase (Figures 4A–F, Supplementary Figure S4).

Regulation of the Wnt/β-catenin signaling pathway by gain-of-function or loss-of-function of NONRATG001910.2.

**FIGURE 5:**
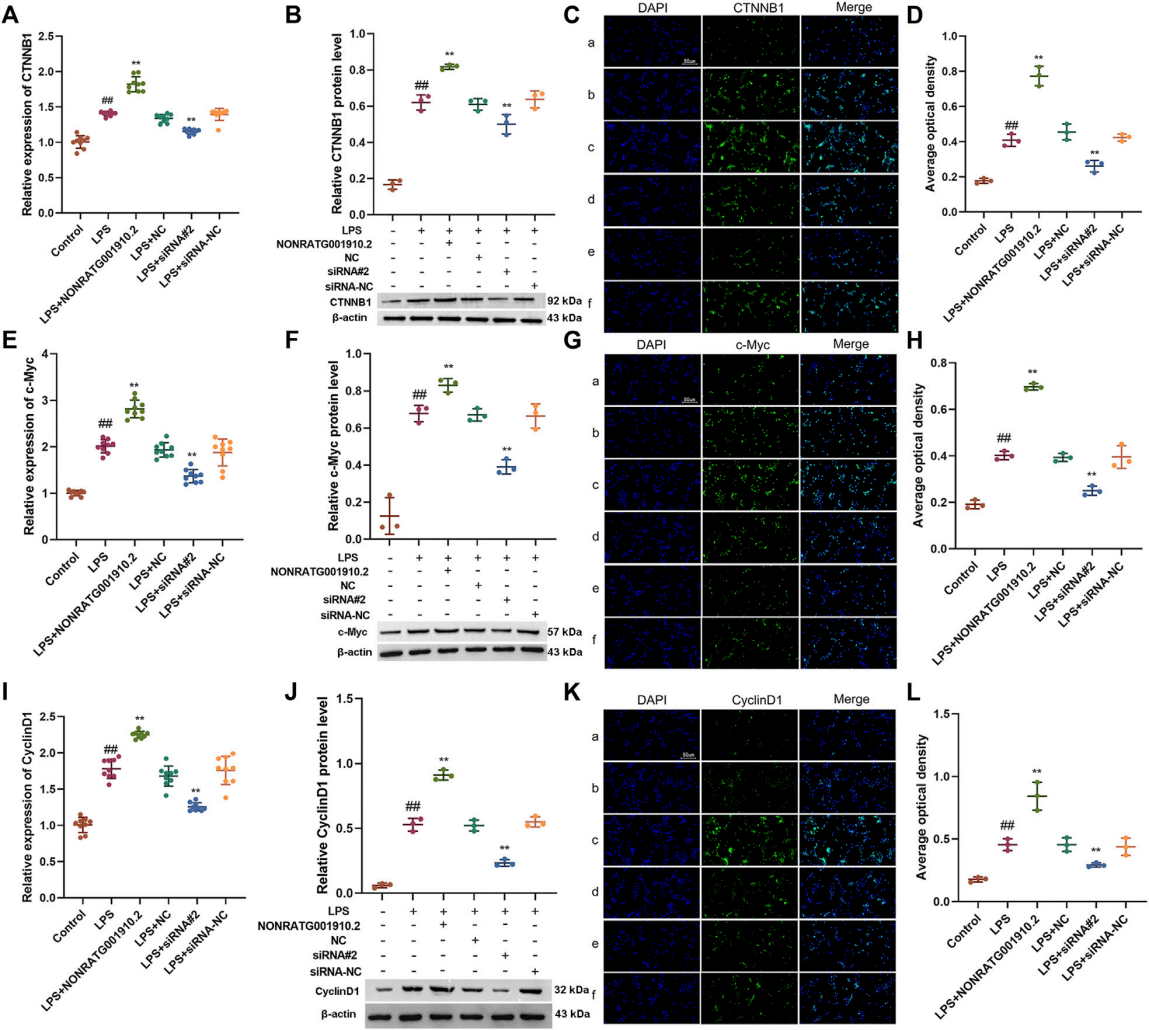
Regulation of the Wnt/β-catenin signaling pathway by gain-of-function or loss-of-function of NONRATG001910.2. **(A–D)** Changes of β-catenin protein and mRNA expression in LPS stimulated HBZY-1 cell lines after gain-of-function or loss-of-function of NONRATG001910.2. **(E–G)** Changes of c-Myc protein and mRNA expression in LPS stimulated HBZY-1 cell lines after gain-of-function or loss-of-function of NONRATG001910.2. **(H–J)** Changes of CyclinD1 protein and mRNA expression in LPS stimulated HBZY-1 cell lines after gain-of-function or loss-of-function of NONRATG001910.2. (a) Control; (b) LPS; (c) LPS + NONRATG001910.2; (d) LPS + NC; (e) LPS + siRNA^#^2; (f) LPS + siRNA-NC. ^##^ *p* < 0.01, compared with the control group. ^**^ *p* < 0.01, compared with the LPS stimulated group.

We found that gain-of-function or loss-of-function of NONRATG001910.2 in LPS-stimulated HBZY-1 cells affected the levels of target genes downstream of the Wnt/β-catenin signaling pathway, including CTNNB1(β-catenin), c-Myc and CyclinD1. With the NONRATG001910.2 knockdown, both mRNA and protein levels of β-catenin in HBZY-1 cells were downregulated. Conversely, the results showed that overexpression of NONRATG001910.2 resulted in the upregulation of β-catenin in mRNA and protein levels (Figures 5A–D). The expression of c-Myc and Cyclin D1 in HBZY-1 cells was significantly reduced after knockdown of NONRATG001910.2. In contrast, overexpression of NONRATG001910.2 enhanced the expression of these genes (Figures 5E–L). Overall, NONRATG001910.2 promotes CGN progression and development through miR-339-3p-mediated regulation of the Wnt/β-catenin signaling pathway.

## Discussion

LncRNAs are considered to be important regulators of biological processes by regulating gene expression at the transcriptional, post-transcriptional and epigenetic levels (Ge et al., 2019; Zhou et al., 2020). In recent years, aberrant dysregulation of lncRNA activation has been elucidated to be involved in disease onset and progression, and more and more lncRNAs are coming into the limelight (Shen et al., 2019; Li J. et al., 2020). Up to now, more and more lncRNAs have been identified. Only a small portion of lncRNAs have been identified to have functional characteristics. However, the role of NONRATG001910.2 in CGN is still largely unknown. Therefore, this study started with NONRATG001910.2 to explore whether it is involved in CGN progression. In the present study, we found a new CGN related NONRATG001910.2, which was found significantly overexpressed in LPS-stimulated HBZY-1 cell lines. Further, cell function assays showed that NONRATG001910.2 promotes the proliferation of HBZY-1 cells and reduces the number of cells remaining in G2 phase, suggesting that NONRATG001910.2 is closely associated with CGN. Our data demonstrated that NONRATG001910.2 takes part in the development and progression of CGN and may be a potential therapeutic target.

According to previous reports, correct lncRNA subcellular localization is essential for biological function (Alessio et al., 2019; Zhang Y. et al., 2020). Growing evidence that lncRNAs in the cytoplasm can act as ceRNA that act as molecular sponges for miRNA to reduce the expression and activity of target miRNA (Yang et al., 2018). In this study, we found that NONRATG001910.2 was preferentially localized in the cytoplasm of LPS-induced HBZY-1 cell lines by FISH and Nucleocytoplasmic fractionation assays. This suggests that it has the potential to function as a miRNA sponge. Dual luciferase results indicated the presence of miR-339-3p binding sites in the NONRATG001910.2 sequence. qRT-PCR assays showed that NONRATG001910.2 was negatively correlated with miR-339-3p in LPS-induced HBZY-1 cells, partially reversing NONRATG001910.2 over-expression mediated cell proliferation. So far, the present study suggests that NONRATG001910.2 may function as a ceRNA through sponge miR-339-3p in LPS-induced HBZY-1 cells.

CTNNB1 gene, also known as β-catenin (Xie et al., 2018; Zhang L. et al., 2020). β-catenin is a key growth stimulator in the Wnt/β-catenin pathway and is associated with cell proliferation, invasion and differentiation (Naseri et al., 2018; Wen et al., 2018). In the present study, we found that CTNNB1 expression was significantly inhibited when NONRATG001910.2 was knocked down in LPS-induced HBZY-1 cells. We speculate that low expression of NONRATG001910.2 lost its sponge effect on miR-339-3p, further leading to β-catenin mRNA degradation. In contrast, in HBZY-1 cells after LPS induction, transfer of NONRATG001910.2 over-expression plasmid resulted in high expression of NONRATG001910.2, miR-339-3p was adsorbed and lost its effect on target genes, and β-catenin expression was released, promoting cell proliferation with fewer cells remaining in the G2 phase, while activating expressions of downstream pathway-related genes, such as CyclinD1 and c-Myc. Dual luciferase results indicated the presence of miR-339-3p binding sites in the CTNNB1 sequence. Analysis of qRT-PCR and WB experimental results showed that CTNNB1 was negatively correlated with miR-339-3p in LPS-induced HBZY-1 cells. In summary, NONRATG001910.2 and miR-339-3p may form a regulatory axis that plays an important regulatory role in the progression of CGN.

**FIGURE 6:**
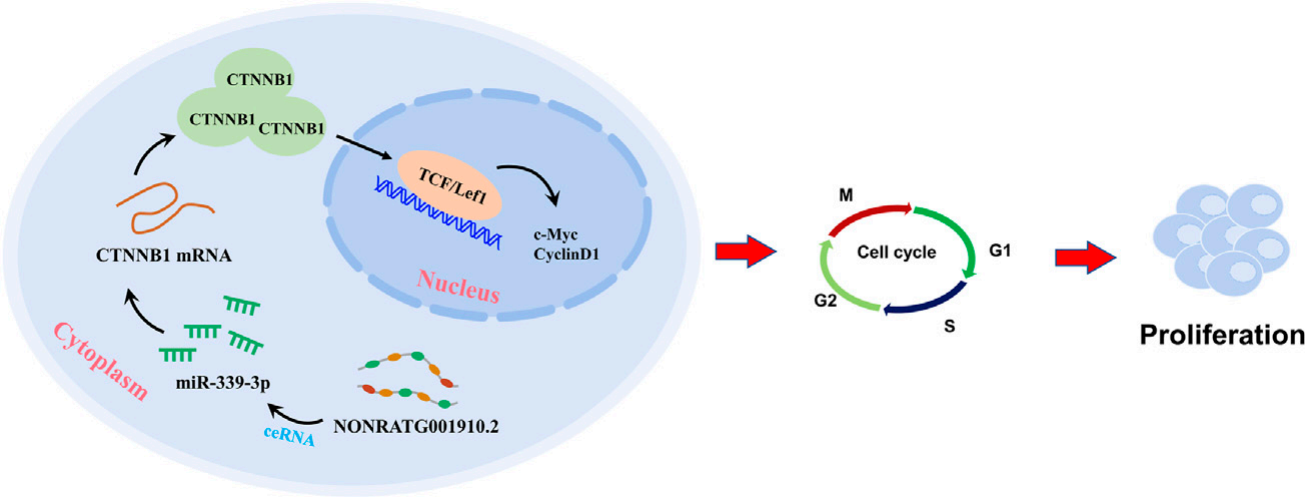
A graphical abstract was created to illustrate the core of this study.

In conclusion, NONRATG001910.2 serves as a ceRNA of miR-339-3p in up-regulating the expression of CTNNB1, thus promoting chronic glomerulonephritis progression (Figure 6). Our study suggests that NONRATG001910.2 may promote the expression of CTNNB1 through the ceRNA pathway and thus affect cell function.

## Data Availability

The original contributions presented in the study are included in the article/Supplementary Material, further inquiries can be directed to the corresponding author.
