# Supplementary material for: Long Non-Coding NONRATG001910.2 Promotes the Proliferation of Rat Mesangial Cell Line HBZY-1 Through the miR-339-3p/CTNNB1 Axis
Source: Front Genet. 2022 Apr 28;13:834144. doi: 10.3389/fgene.2022.834144 (PMC9096093; doi:10.3389/fgene.2022.834144)
Supplement: Supplementary file 1 [file Table1.DOCX]

**Supplementary Table 1. Primers for qRT-PCR**

| **Gene name** | **Forward primer (5’-3’)** | **Reverse primer (5’-3’)** |
| --- | --- | --- |
| NONRATG001910.2 | GTCCACAGCCTCACGCCTAC | AGTTCAGTCTGGGTTCGTTCCT |
| miR-339-3p | ACACTCCAGCTGGGTGAGCGCCTCGACGACA | TGGTGTCGTGGAGTCG |
| β-catenin | GACCCCAAGCCTTAGTAAACA | GACAGACAGCACCTTCAGCA |
| c-Myc | GCGAGAACAGTTGAAACACA | GGAACCGTTCTCCTTACACT |
| CyclinD1 | TCAAGTGTGACCCGGACTG | GACCAGCTTCTTCCTCCACTT |
| β-actin | CCCATCTATGAGGGTTACGC | TTTAATGTCACGCACGATTTC |
| U6 | CTCGCTTCGGCAGCACA | AACGCTTCACGAATTTGCGT |
