# Supplementary material for: Long Non-Coding NONRATG001910.2 Promotes the Proliferation of Rat Mesangial Cell Line HBZY-1 Through the miR-339-3p/CTNNB1 Axis
Source: Front Genet. 2022 Apr 28;13:834144. doi: 10.3389/fgene.2022.834144 (PMC9096093; doi:10.3389/fgene.2022.834144)
Supplement: Supplementary file 2 [file DataSheet1.ZIP › Abbreviations.docx]

Abbreviations

CGN, chronic glomerulonephritis; ESRD, end-stage renal disease; lncRNAs, long non-coding RNAs; qRT-RCR, quantitative real-time polymerase chain reaction; HBZY-1, rat mesangial cell line HBZY-1; CKD, chronic kidney disease; ncRNA, non-coding RNA; LPS, lipopolysaccharide; miR-NC, microRNA-negative control; siRNA, small interfering RNA against NONRATG001910.2; siRNA-NC, siRNA-negative control; microRNA-339-3p mimic, miR-339-3p mimic; miR-339-3p inhibitor, microRNA-339-3p inhibitor; NONRATG001910.2, NONRATG001910.2 overexpression plasmid; NC, negative control; siCTNNB1, small interfering RNA against CTNNB1; siCTNNB1-NC, siCTNNB1-negative control; mRNA, messenger RNA.
