# Supplementary material for: Long Non-Coding NONRATG001910.2 Promotes the Proliferation of Rat Mesangial Cell Line HBZY-1 Through the miR-339-3p/CTNNB1 Axis
Source: Front Genet. 2022 Apr 28;13:834144. doi: 10.3389/fgene.2022.834144 (PMC9096093; doi:10.3389/fgene.2022.834144)
Supplement: Supplementary file 2 [file DataSheet1.ZIP › Supplementary Figure.docx]

Supplementary Figure
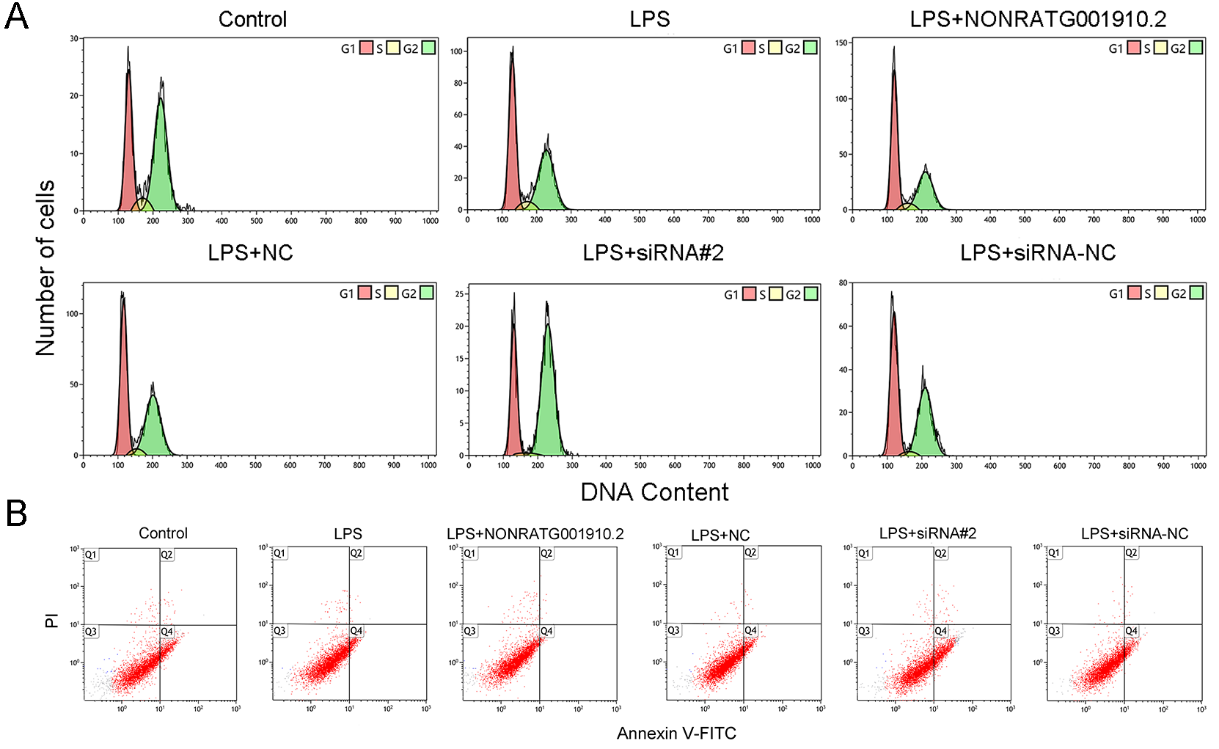
1 Flow cytometric detection of cell cycle distribution (A) and apoptosis (B) in HBZY-1 cells in the gain-of-function or loss-of-function state of NONRATG001910.2.


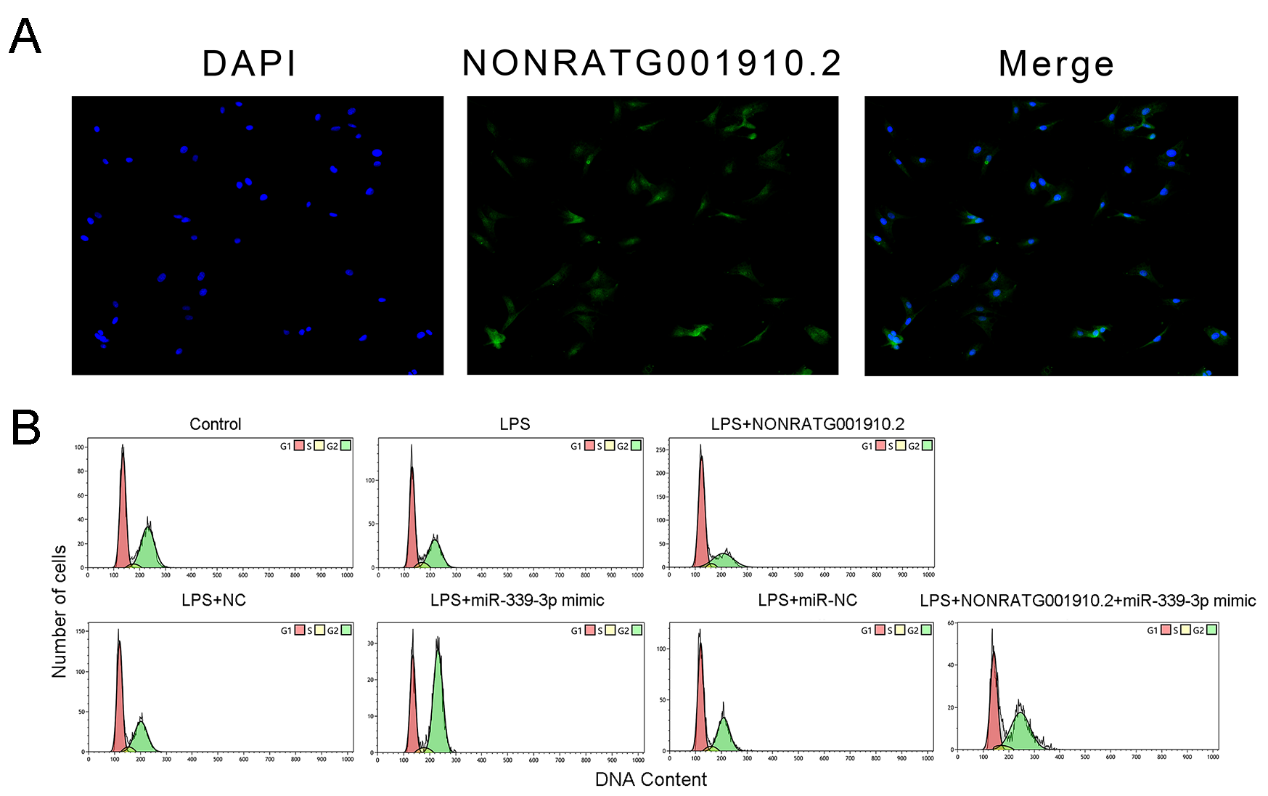


Supplementary Figure 2 (A)FISH assays showing the location of NONRATG001910.2 in HBZY-1 cells. and apoptosis (B) Flow cytometric detection of cell cycle distribution.


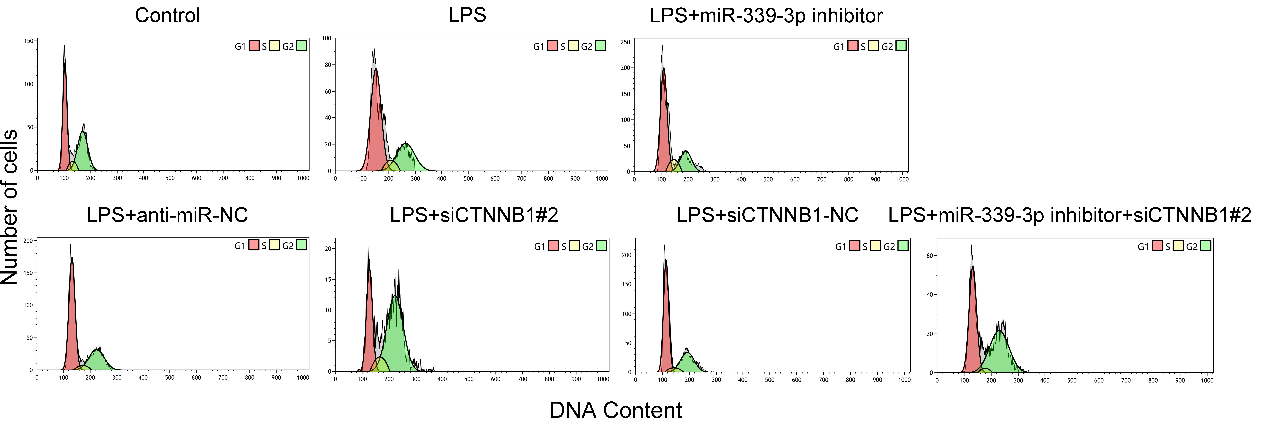


Supplementary Figure 3 Flow cytometric detection of cell cycle distribution.


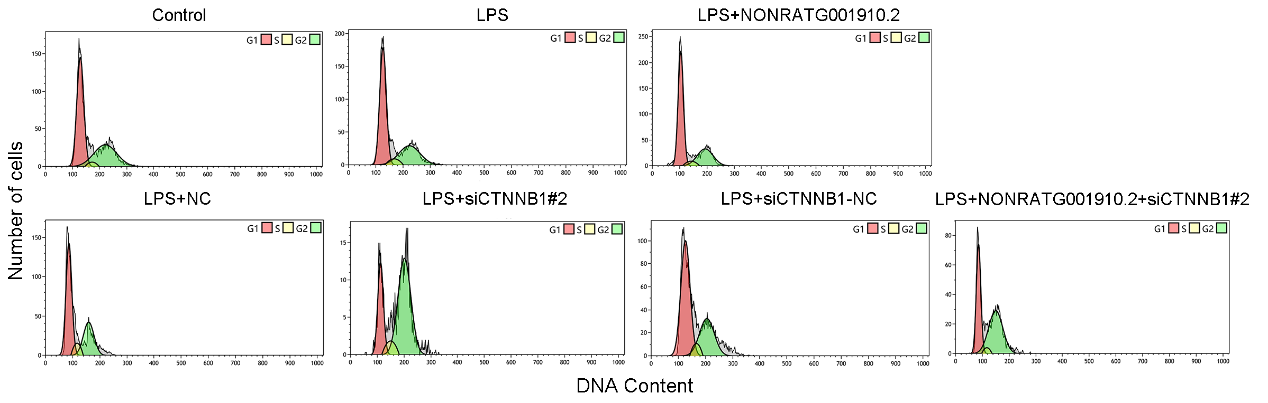


Supplementary Figure 4 Flow cytometric detection of cell cycle distribution.
