# Supplementary figures and images for: Long Non-Coding NONRATG001910.2 Promotes the Proliferation of Rat Mesangial Cell Line HBZY-1 Through the miR-339-3p/CTNNB1 Axis
Source: Front Genet. 2022 Apr 28;13:834144. doi: 10.3389/fgene.2022.834144 (PMC9096093; doi:10.3389/fgene.2022.834144)

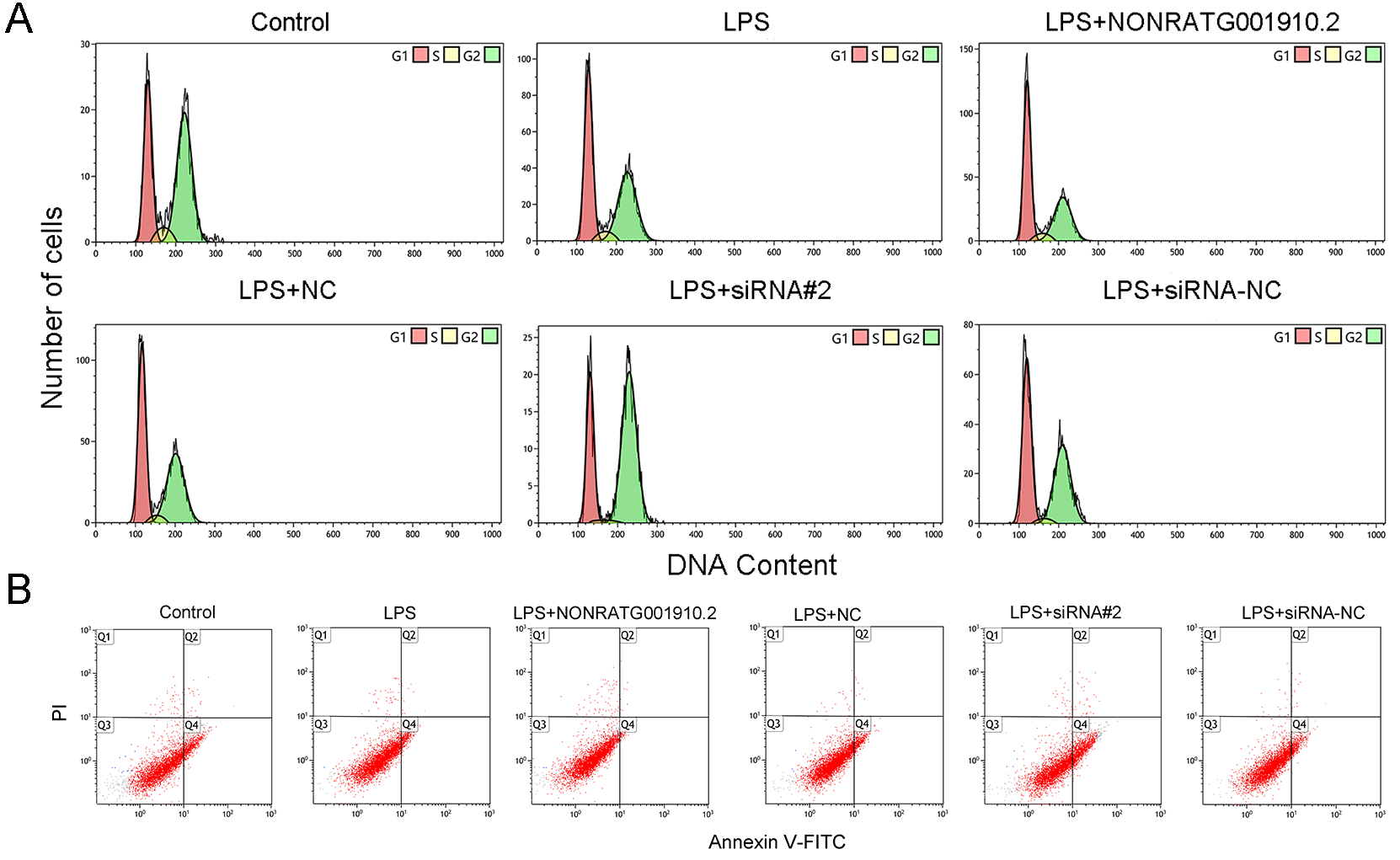

Supplement: Supplementary file 2 [file DataSheet1.ZIP › Supplementary Figure 1.tif]

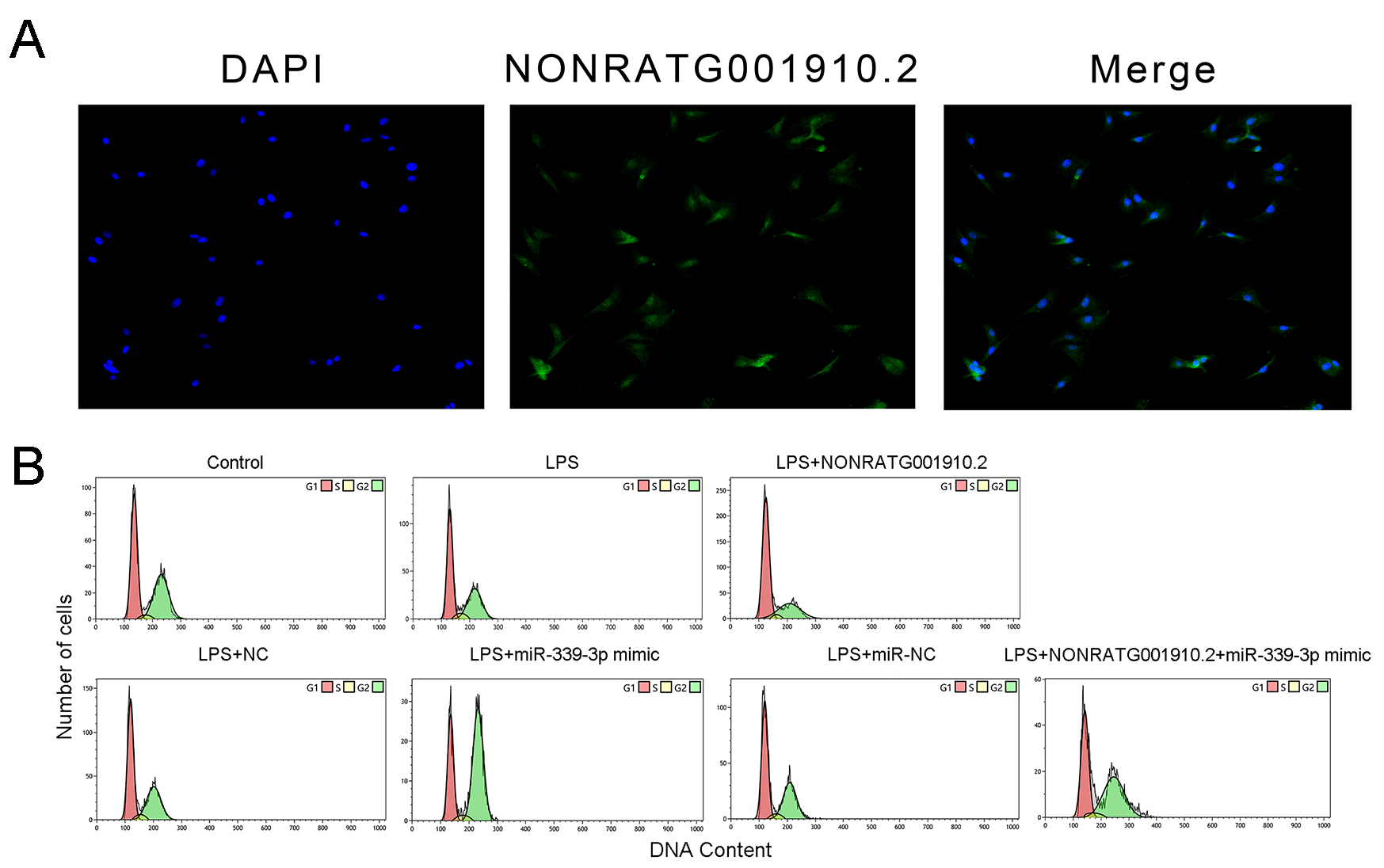

Supplement: Supplementary file 2 [file DataSheet1.ZIP › Supplementary Figure 2.tif]

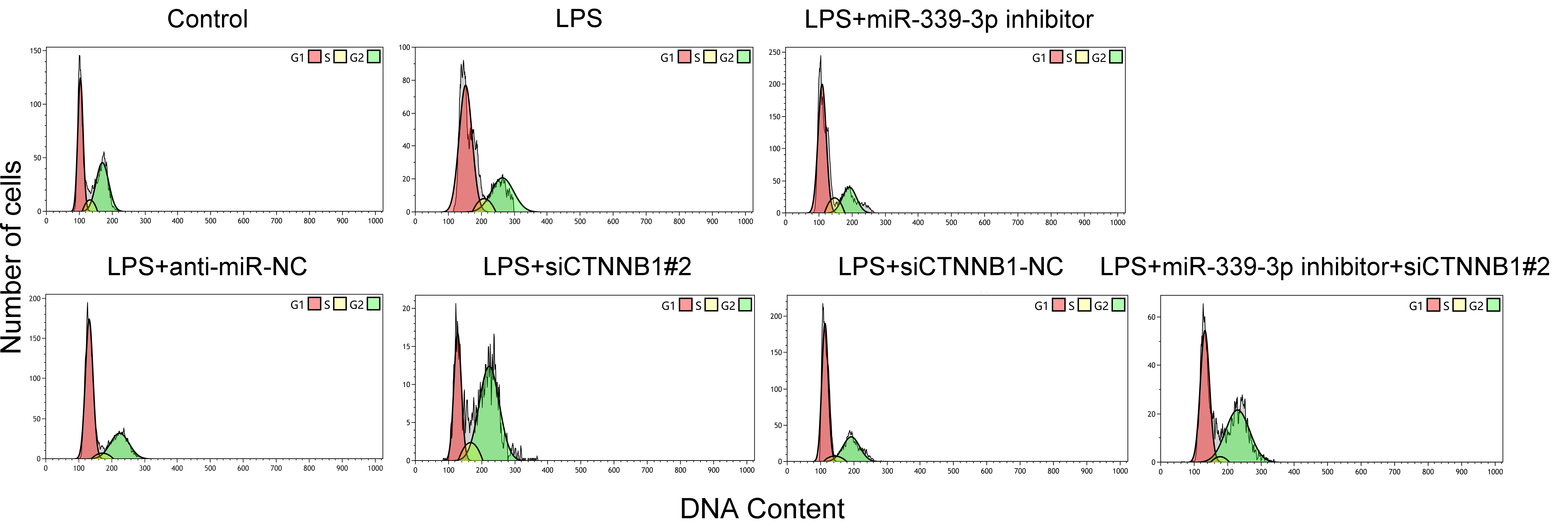

Supplement: Supplementary file 2 [file DataSheet1.ZIP › Supplementary Figure 3.tif]

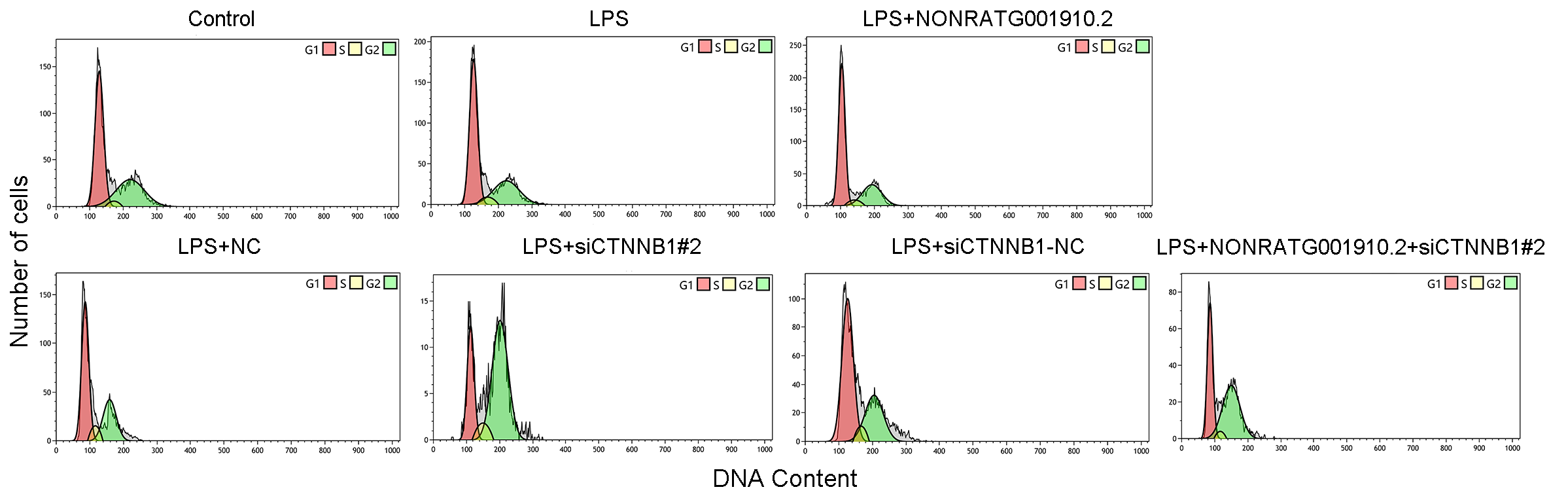

Supplement: Supplementary file 2 [file DataSheet1.ZIP › Supplementary Figure 4.tif]

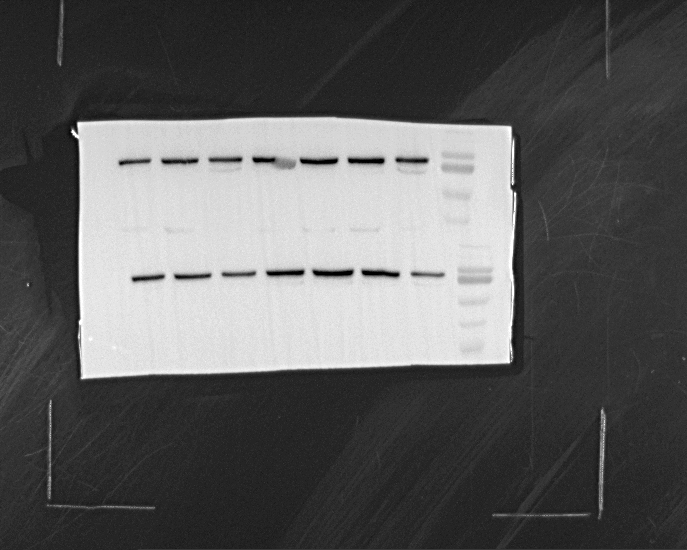

Supplement: Supplementary file 2 [file DataSheet1.ZIP › WB/Figure 3/CTNNB1/CTNNB1 in manuscript(down).tif]

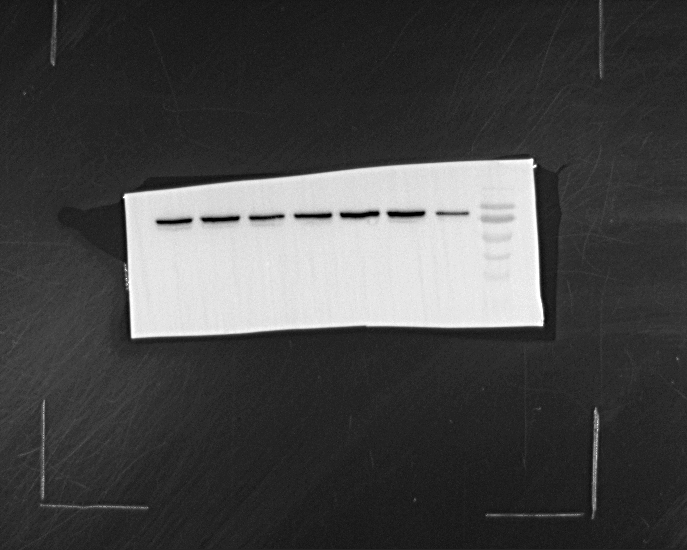

Supplement: Supplementary file 2 [file DataSheet1.ZIP › WB/Figure 3/CTNNB1/CTNNB1-1 .tif]

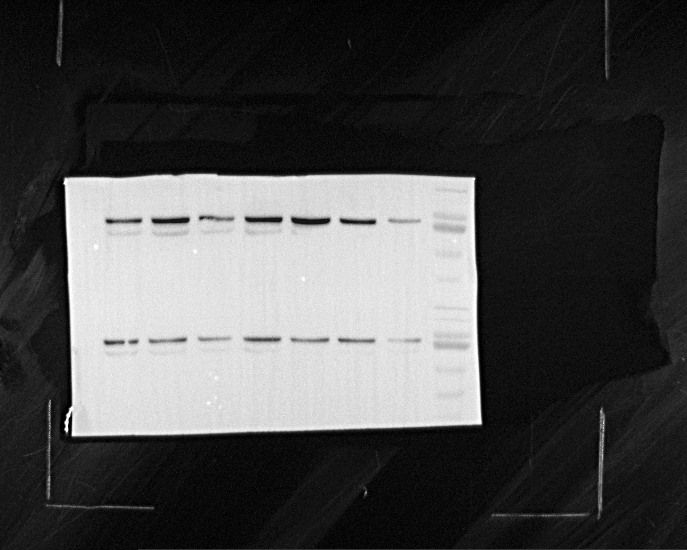

Supplement: Supplementary file 2 [file DataSheet1.ZIP › WB/Figure 3/CTNNB1/CTNNB1-2.tif]

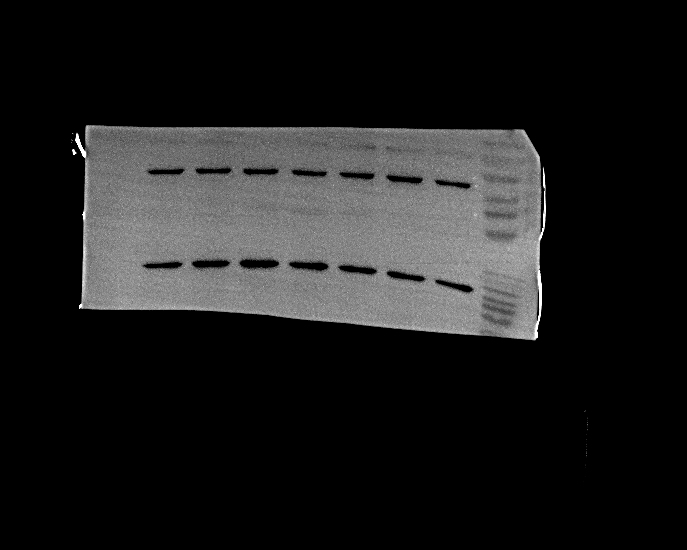

Supplement: Supplementary file 2 [file DataSheet1.ZIP › WB/Figure 3/β-actin/β-actin in manuscript(up) .tif]

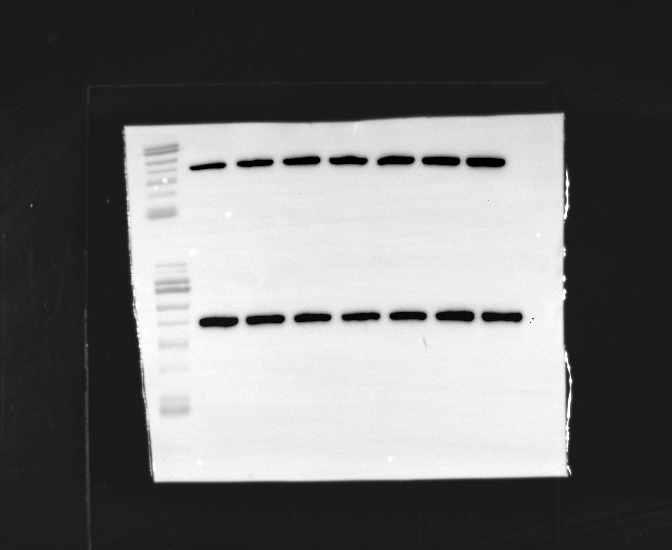

Supplement: Supplementary file 2 [file DataSheet1.ZIP › WB/Figure 3/β-actin/β-actin.tif]

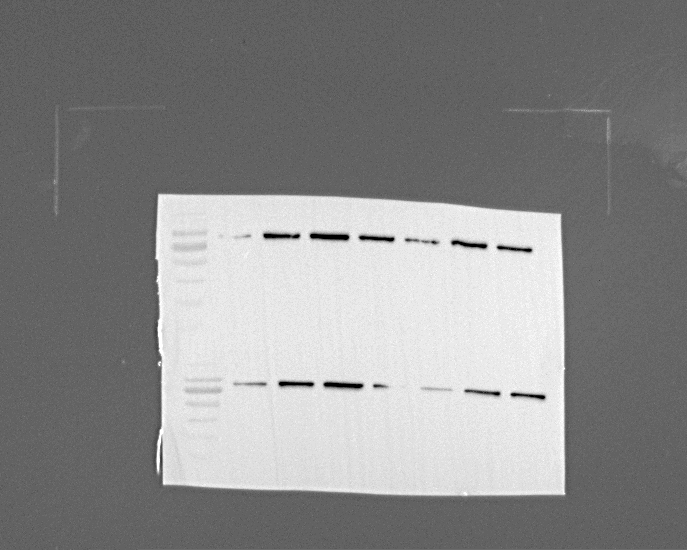

Supplement: Supplementary file 2 [file DataSheet1.ZIP › WB/Figure 4/CTNNB1/CTNNB1 in manuscript(up).tif]

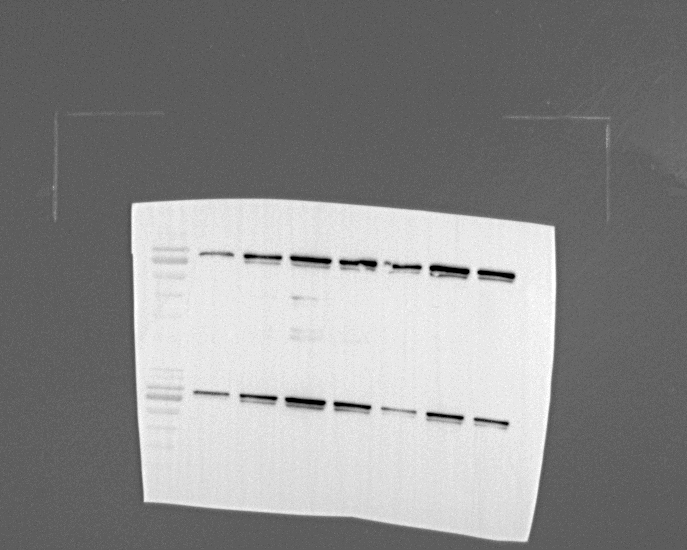

Supplement: Supplementary file 2 [file DataSheet1.ZIP › WB/Figure 4/CTNNB1/CTNNB1.tif]

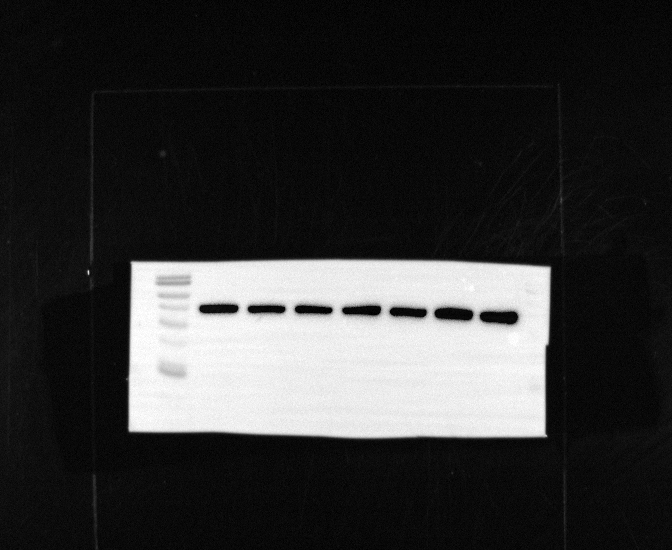

Supplement: Supplementary file 2 [file DataSheet1.ZIP › WB/Figure 4/β-actin/β-actin.tif]

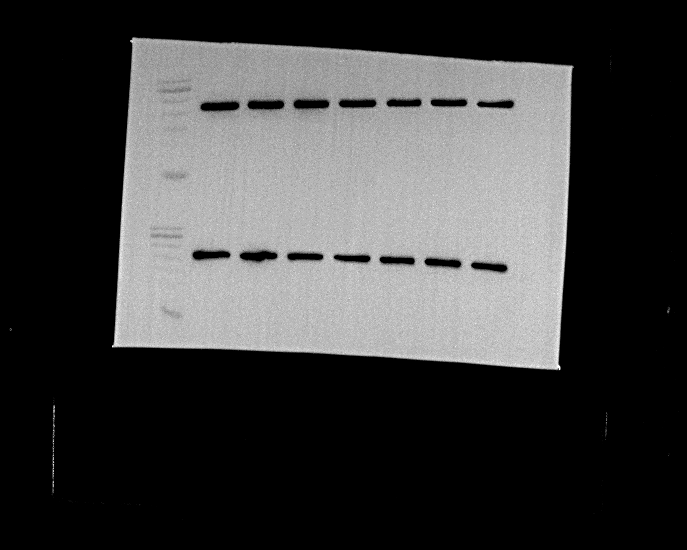

Supplement: Supplementary file 2 [file DataSheet1.ZIP › WB/Figure 4/β-actin/β-ctin in manuscript (down).tif]

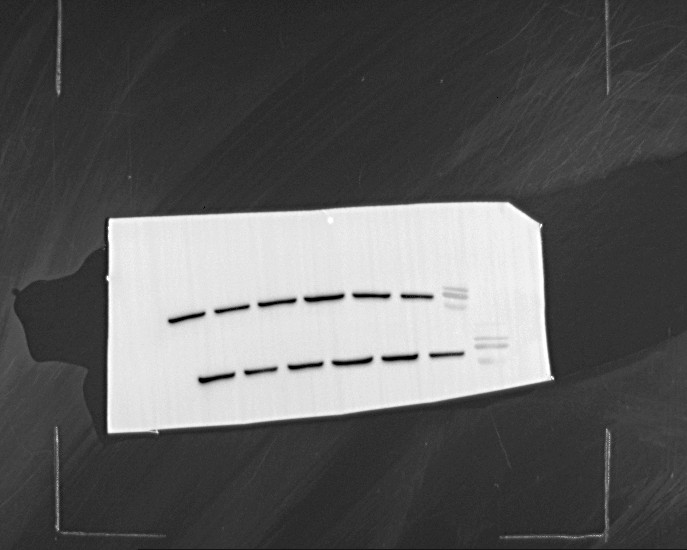

Supplement: Supplementary file 2 [file DataSheet1.ZIP › WB/Figure 5/c-Myc/c-Myc in manuscript(down).tif]

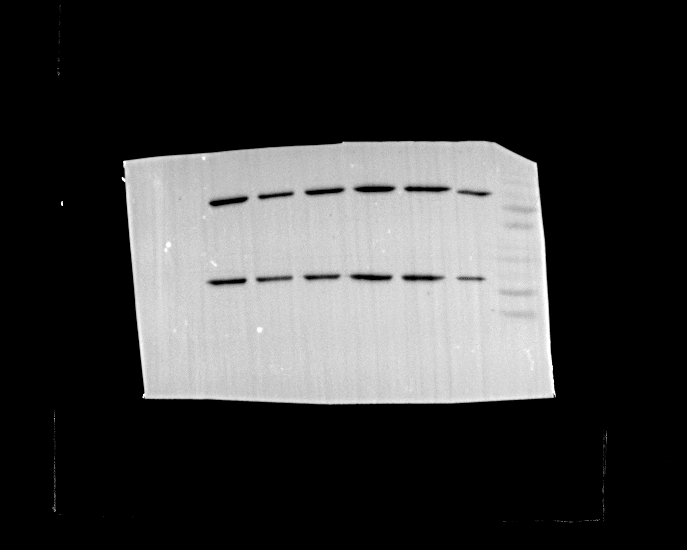

Supplement: Supplementary file 2 [file DataSheet1.ZIP › WB/Figure 5/c-Myc/c-Myc-2.tif]

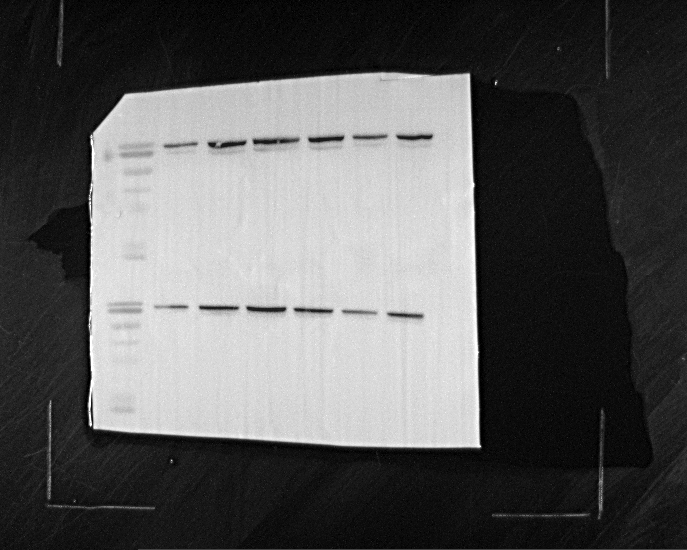

Supplement: Supplementary file 2 [file DataSheet1.ZIP › WB/Figure 5/CTNNB1/CTNNB1 in manuscript(down) .tif]

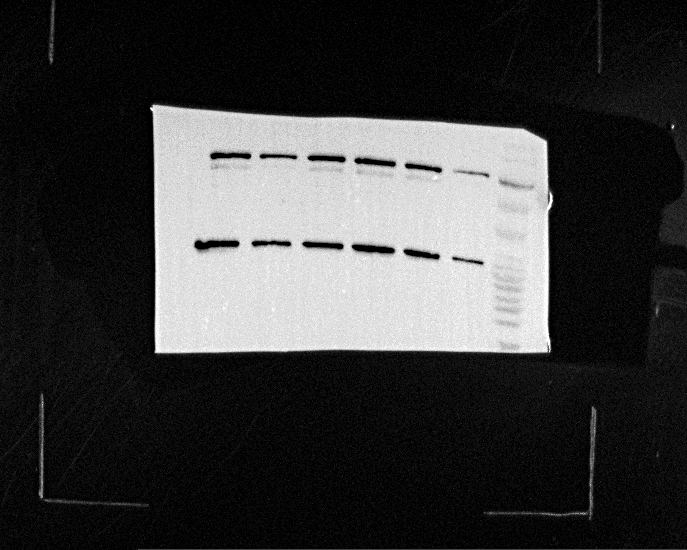

Supplement: Supplementary file 2 [file DataSheet1.ZIP › WB/Figure 5/CTNNB1/CTNNB1.tif]

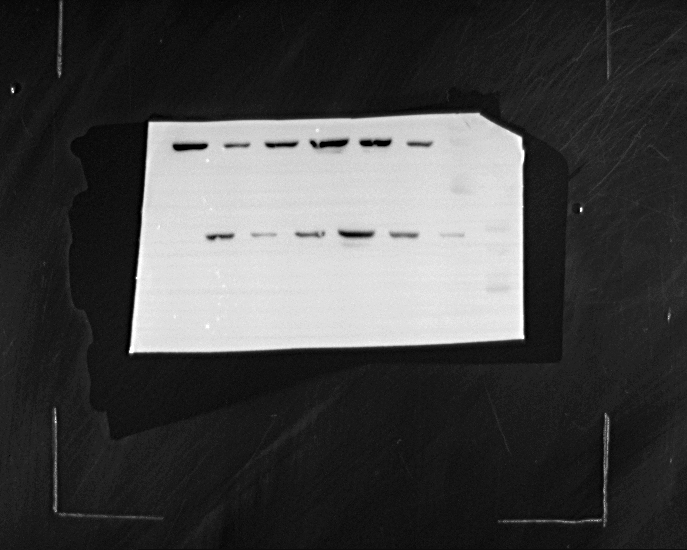

Supplement: Supplementary file 2 [file DataSheet1.ZIP › WB/Figure 5/CyclinD1/Cyclin D1 in manuscript(up).tif]

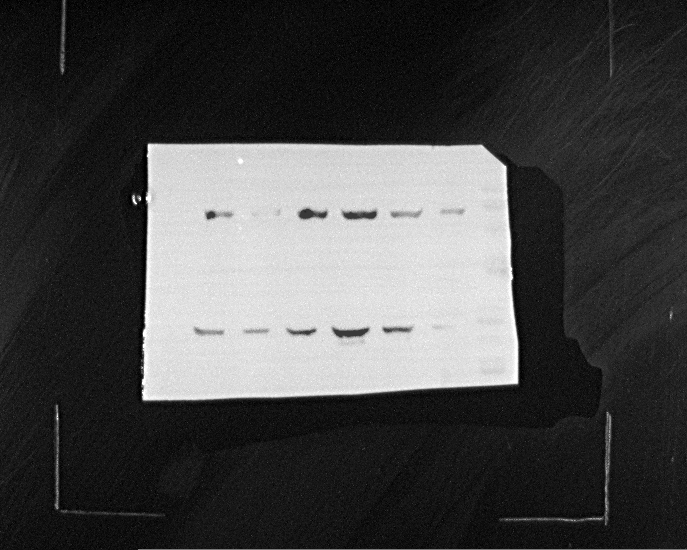

Supplement: Supplementary file 2 [file DataSheet1.ZIP › WB/Figure 5/CyclinD1/Cyclin D1.tif]

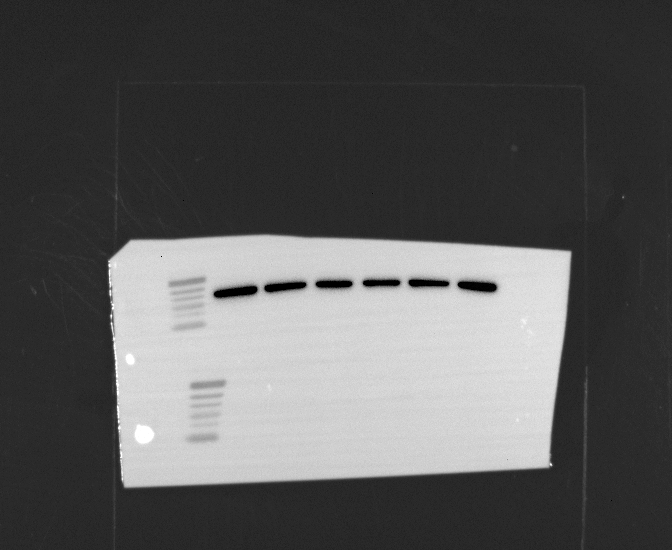

Supplement: Supplementary file 2 [file DataSheet1.ZIP › WB/Figure 5/β-actin/Actin-1.tif]

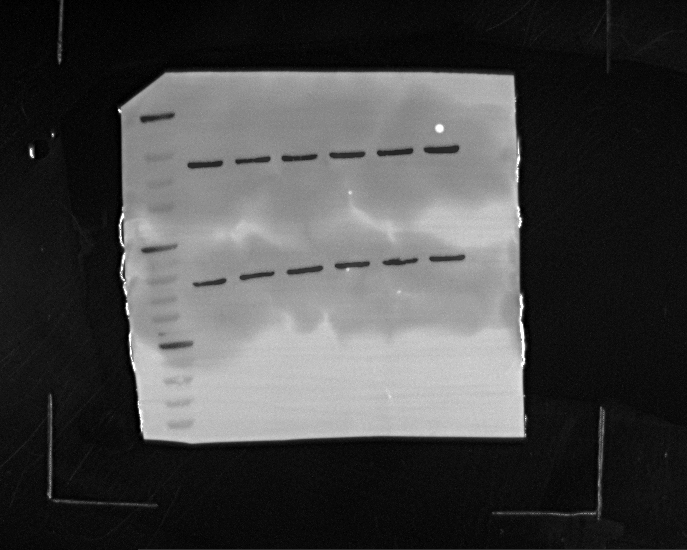

Supplement: Supplementary file 2 [file DataSheet1.ZIP › WB/Figure 5/β-actin/Actin-12&13.tif]

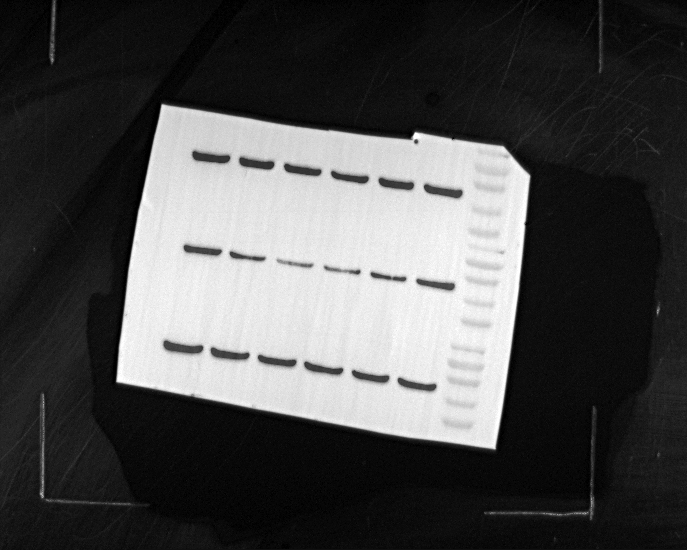

Supplement: Supplementary file 2 [file DataSheet1.ZIP › WB/Figure 5/β-actin/Actin-14.tif]

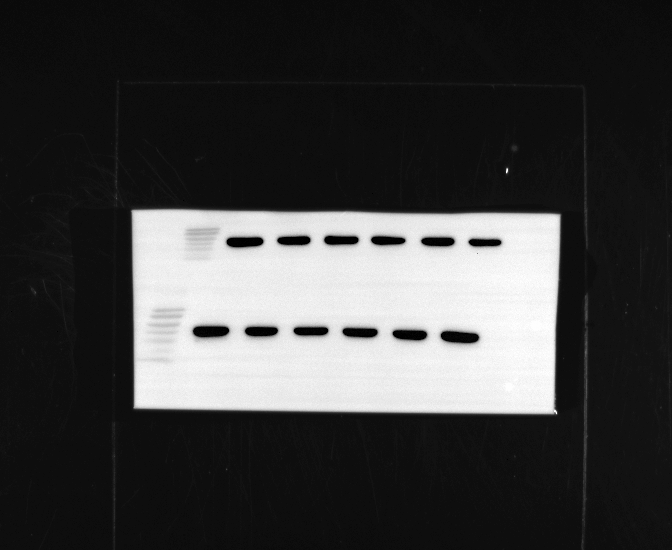

Supplement: Supplementary file 2 [file DataSheet1.ZIP › WB/Figure 5/β-actin/Actin-2&3.tif]

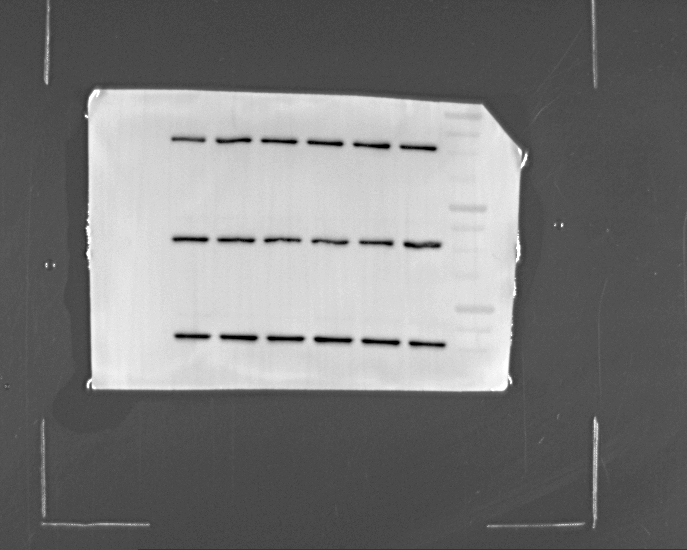

Supplement: Supplementary file 2 [file DataSheet1.ZIP › WB/Figure 5/β-actin/Actin-7&8.tif]

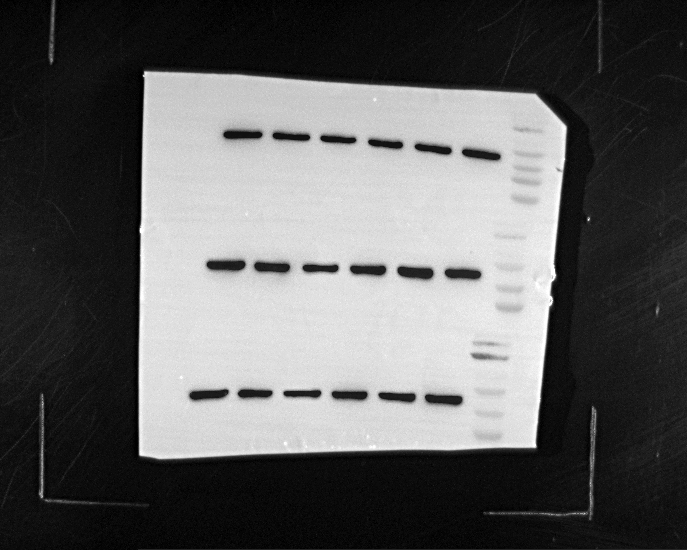

Supplement: Supplementary file 2 [file DataSheet1.ZIP › WB/Figure 5/β-actin/Actin-c-Myc in manuscript(up).tif]

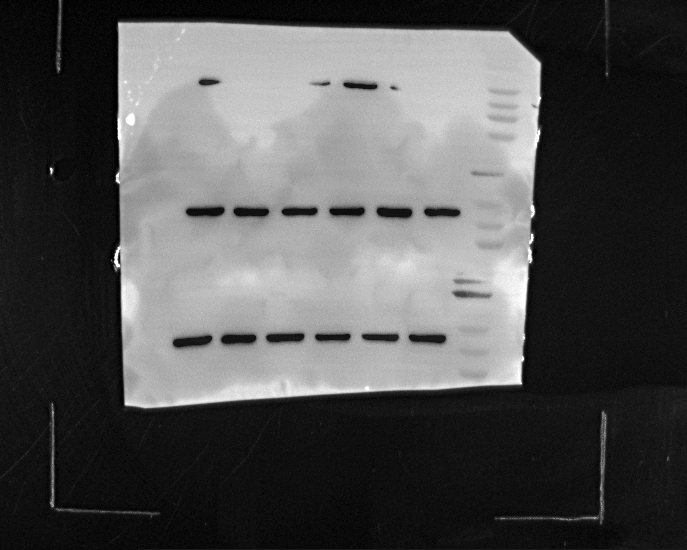

Supplement: Supplementary file 2 [file DataSheet1.ZIP › WB/Figure 5/β-actin/Actin-CyclinD1 in manuscrip(middle).tif]
